# Experiences and Lessons from Agri-Food System Transformation for Sustainable Food Security: A Review of China’s Practices

**DOI:** 10.3390/foods11020137

**Published:** 2022-01-06

**Authors:** Yujia Lu, Yongxun Zhang, Yu Hong, Lulu He, Yangfen Chen

**Affiliations:** 1 Institute of Agricultural Economics and Development, Chinese Academy of Agricultural Sciences, Beijing 100081, China; 82101201652@caas.cn (Y.L.); zhangyongxun@caas.cn (Y.Z.); hongyu@caas.cn (Y.H.); 2 College of Humanities and Development, China Agricultural University, No. 2 Yuanmingyuan West Road, Haidian District, Beijing 100094, China; helulu@cau.edu.cn

**Keywords:** agri-food system, food security, transformation, China, sustainability

## Abstract

Food system transformation has been a widely discussed topic in international society over time. For the last few decades, China has made remarkable achievements in food production and has contributed greatly to the reduction in global hunger and poverty. Examining experiences and lessons from China’s food security practices over the years is helpful to promote a national food system transformation for China, as well as other developing countries. This study systematically reviews the literature on Chinese food security studies, with the aim of assessing China’s food security achievements and examining the remaining and emerging issues in the pursuit of food system transformation. The results show that China has continuously promoted food system transformation in land consolidation, agri-food production technologies, management and organization modes, food reserves, trade governance, and food consumption. These transformations ensure not only food availability, timeliness, and nutrition, but also in terms of the ecological, social, and economic sustainability, feasibility, and justice of food security. However, China is also confronting new challenges in food security, for example, malnutrition, environmental unsustainability, and reductions in diversified agri-food. In the future, China is expected to be committed to promoting healthy diets, sustainable agricultural production, climate change mitigation, and the reduction of food waste and loss to enhance its agri-food system’s resilience.

## 1. Introduction

Achieving the goal of global food security, together with environmental sustainability and socio-economic justice, is one of the greatest challenges for human beings in the 21st century [1]. There are several factors impeding progress toward achieving global food security and nutrition targets. For instance, starting from 2019, it is estimated that the outbreak of the COVID-19 pandemic has led to an increase in undernourishment from 8.4 to 9.9% worldwide, leaving nearly 2.37 billion people without access to adequate food supplies, while 2.2 billion adults are overweight [2,3,4]. Human activities directly affect more than 70% of global ice-free land at present, and up to one-third of the terrestrial net primary productivity is being used for food, feed, fiber, timber, and energy [5]. Food systems that rely heavily on chemical fertilizers, pesticides, and antibiotics [6] have driven the widespread degradation of land, water and ecosystems, high greenhouse gas (GHG) emissions, biodiversity losses [7], and the spread of pathogens [8], which in turn lead to food insecurity in many countries [9]. It is projected that climate change will cause about 250,000 deaths each year, resulting from malnutrition, malaria, diarrhea, and heat stress, between 2030 and 2050 [10].

Food security itself is a multi-dimensional concept, with goals ranging from ensuring survival and health to gradually considering food preferences, involving food accessibility, availability, utilization, and stability [11], and it operates within social, economic, environmental, and political contexts [12]. It is inconclusive how analysts should rank the various indicators that influence food security [13], which can only be achieved when the specific objective is clearly stated [14]. It will be more valuable for policy guidance to identify clearly with food security’s meaning, limitations, and interaction approaches with non-food factors, from a systematic perspective [15]. Globally, the in-depth structural transformation of agri-food systems is expected to be achieved in the future in a more inclusive way [1], i.e., delivering healthy food for human beings, and that it should be resilient to shocks from climate change, economic risk, and market failures [16].

Current trends in poverty, malnutrition, climate change and economic turmoil have driven global food insecurity [17], suggesting widespread failures in food systems [1]. The COVID-19 pandemic continues to expose the weaknesses of current global food systems and heighten concerns over food security risks, especially in low- and middle-income countries [18]. It has been observed that the evolution of our food systems should shift from exclusively boosting production to nourishing people in a more inclusive and sustainable approach, to ensure that future generations will be better provided for [19]. As food system transformation is expected to reflect the approach of pursuing social, environmental, nutritional and health outcomes and potential influences, ensuring global food security to achieve the Sustainable Development Goals will depend on the potential of food systems to increase the efficiency and resilience of the food supply chain.

The ability to deliver affordable and nutritional food and the resilience to adapt to a new normal of regional food systems varied considerably as a result of numerous interacting factors, such as agricultural resources, socio-economic context, and policy orientation [20]. As mentioned earlier, China has made tremendous progress in reducing hunger and food insecurity in recent decades. The total number of undernourished people in China fell from 289 million in 1990–1992, to 125 million in 2016–2017 [21]. Chinese food systems may have global implications, as the country is the largest producer, importer, and exporter of many food commodities, and is the biggest GHG emitter from agri-food systems [22]. More importantly, China has increasingly become a key global player and is an increasingly influential actor in global agri-food network governance, through participating in world cooperation, initiatives, and activities [23]. China and India are considered the countries that are best equipped to instigate food system changes, because they have the necessary “technological capacities, state-regulatory systems and socio-economic need” [24]. Therefore, it would be valuable for other developing countries to analyze the experience of China’s food system transformation, although there is no one-size-fits-all solution for all countries. Policymakers will need to assess their own context-specific challenges to achieve the required transformations [25].

**Figure 1:**
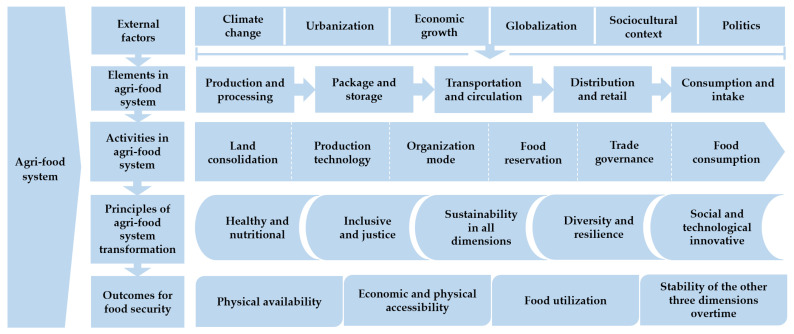
Research framework.

Considering a food system captures all the elements and activities that relate to the food supply chain and the outputs of these activities, including the socio-economic and environmental outcomes [26] (Figure 1). This paper will systematically review China’s experiences from the perspective of the food supply chain, including food production and management, food reserves, food trade and circulation, as well as food demand and consumption, and then summarize the challenges it faces, the achievements and outcomes of food security in China. It is expected to provide details of real experiences and lessons for global poverty reduction and malnutrition elimination in developing countries.

## 2. Materials and Methods

We conducted extensive literature reviews constituting four steps: identification, screening, eligibility, and inclusion [27], in order to identify peer-reviewed journal articles and gray literature documents that reported the transformation of the food system in China. At the identification step, in the WOS Core Collection, we set the themes as “food security”, “food system”, and “China”. In total, 1323 documents were found, of which 469 belonged to the environmental sciences category. Then, by resetting the themes as “food security”, “food system transformation” and “China”, 43 documents were found. In the CNKI-CSSCI collection, we set the themes as “food security” and “food system”, resulting in 36 documents. For the sake of the comprehensiveness of the included body of literature [28], grey literature was retrieved by searching Google Scholar and the websites of five organizations, including the UN Food and Agriculture Organization (FAO), the International Panel of Experts on Sustainable Food Systems (IPES-Food), the Intergovernmental Panel on Climate Change (IPCC), the World Health Organization (WHO), and the Global Nutrition Report. The objective of this paper was not specifically to assess the effect of China’s food policy, but instead to analyze the experience of China’s food system transformation from the perspective of the supply chain, including both policy innovation and informal trends, and to obtain enlightenment on global food security governance, including experience and lessons. During the screening and eligibility steps, we reviewed the titles and abstracts of identified articles and examined citations from documents for additional references, after which 96 articles were retained. At the inclusion step, the latter articles were carefully reviewed and all the results presented in this study are based on the analysis of these 96 articles.

## 3. Results

### 3.1. China’s Food Security Challenges

#### 3.1.1. Increasing Pressure on Resources

Firstly, the deterioration in the quantity and quality of natural resources in China has been attributed to the main constraints on food production. According to the third national land survey, the total area of cultivated land in China was about 0.13 billion ha in 2019, which is 7.53 million ha less than that in 2013. The per capita of arable land decreased from 0.106 ha in 1996 to 0.085 ha in 2018, approximating 46.3% of the world’s average. Regarding the current state of land productivity, it was reported that high-quality farmland and reserve land have little room to improve in terms of productivity, while there have been increasing restrictions on low- and medium-level field transformation. These changes have increased concerns about the future development of agricultural production [29]. Secondly, population growth, rising household incomes, and changing dietary patterns will contribute to an increase in the demand for food. With the two-child policy scenario in China, it is estimated that food demand will reach its peak at 39.26 million tons by 2030 [30]. The food consumption pattern in China has gradually transformed from a vegetable-based dietary structure to an animal-based one [31,32]. With the expansion of urbanization, Chinese meat consumption is anticipated to arrive at its peak of 123.44 million tons in 2030, leaving great scope for growth in demand for dairy and aquatic products [33].

#### 3.1.2. Smallholder Vulnerability

Small-scale farmers still play a critical role in reducing rural poverty and ensuring national food and nutrition security [34]. One problem is that with China’s aging population, many formerly rural laborers have moved to urban areas, and most rural areas face prominent non-agricultural livelihood transformations, leaving questions such as: “Who will cultivate the land in China?”, exerting a negative impact on the domestic food supply [35,36]. In 2018, 59.3% of the rural employed population were engaged in primary industry. Since 2015, the wage incomes of farmers have exceeded the operating income, accounting for 41% of the total income in 2018 [37]. Another issue is that resources are re-allocated from low-profit industries (agriculture) to high-profit industries [38]. The average cost of cultivating crops (16633.5 yuan/ha) in 2019 was 2.2 times above that in 2001; the average net profit was a negative value from 2016 of minus 457.5 yuan/ha in 2019 [39]. In addition, global socio-economical change, and low levels of adaptive capacity caused by inequality and poverty, will exacerbate the challenges that smallholders already face [40].

#### 3.1.3. Increasing Ecological Impact

Ecological problems resulting from the misallocation of resources and food loss in the supply chain are increasingly prominent in China. On the one hand, the rapid changes in grain production patterns in China have increased the vulnerability of the food system. The gravity center of China’s grain production has gradually shifted to the north away from the south, and the role of the northern part of China has changed from a major grain-consuming area to a major grain-producing zone [41]. However, water resources in northern regions account for approximately 19% of the total water resources in China, while cultivated land area accounts for 60% [42]; the food provision in Northeast China is approaching its maximum potential capacity. Therefore, it is not wise to rely solely on food provision increases in North China [43], which may increase risks such as water shortages and ecosystem deterioration, and eventually reduce the comprehensive production capacity of grain as well as increase the cost of access to food [33,43]. On the other hand, approximately one-third of the food produced globally for humans is lost or wasted along the food supply chain [44]. Between 2014 and 2018, approximately 27% of China’s total food production was lost or wasted annually, making up about one-quarter of the world’s total food losses [45].

#### 3.1.4. Dynamic Global Agricultural and Food Policies

With the growth of globalization, the depth of the impact of the global economic and trade environment on China’s food security has increased significantly. China’s agricultural imports reached USD 170.8 billion in 2020, a 14-fold increase over that in 2001. The import of grain, cotton, oil, sugar, and milk was equivalent to the output of more than 66.7 million ha of cultivated sown area of agricultural land, accounting for 40% of the total planting area of domestic crops [46]. Of these, soybean imports exceeded 100 million tons in 2020, equivalent to 5.1 times that of domestic production. Global food supply chains expose vulnerability as a result of the high concentration of natural resources, unchangeable marine transportation, and unpredictable international situations. In addition, China’s participation in global food governance is challenged by international society, with opinions such as “neocolonialism” and “land grabbing” being voiced, and domestic policies are strictly bound by international regulations [47]. These challenges work together to limit the feasible space for China to use the international market to ensure food security.

### 3.2. China’s Food Security Practices

#### 3.2.1. Land Consolidation

Rural development has long been the priority of national planning in Chinese policy. Land consolidation is recognized as a positive factor with economic, social, ecological, and psychological effects around the world [24], one that involves land redistribution and infrastructure construction, and that could be an ideal tool to improve the efficiency of land use and the revitalization of rural areas [48,49]. After establishing a system for separating the ownership rights, contract rights, and management rights for contracted rural land, the Chinese government has implemented an overall plan for land use throughout the country. Since 2004, with the implementation of land remediation measures such as “Permanent basic farmland protection”, “Balancing the occupation and replenishment of arable land”, “Building High-Standard Basic Farmland”, and a compensation system for the people responsible for making it work, China has gradually formed a complete system of farmland protection policy, which has basically achieved no reduction in the cultivated land area [50]. Abiding by the principles of basic food self-sufficiency, based on domestic grain production, China practices the strictest farmland protection system and employs a strategy of sustainable farmland use to increase farmland productivity [51]. Recently, China announced the implementation of “Preventing the use of arable land for non-farming purposes” in the 2020 Annual Central Economic Work Conference to protect the soil fertility of cultivated land by means of crop rotation, according to regional climate, crop types and soil characteristics.

Land policy in China is now focused on the trinity of quantity control, quality management, and eco-environmental protection [52]. At the same time, a series of research projects on land consolidation was carried out following policy orientation, including “non-grain” production behavior [53], agricultural layout optimization [54], land-function zoning [55], and spatial feasibility [56]. Most of these studies were aimed at exploring theoretical support and feasibility evaluations for policymaking from diverse perspectives and multi-scale approaches, and recently began to focus on the relationship between land ecology and food security [57,58]; however, most of the studies were limited to describing the subject as well lack specific trade-offs between policy objectives. Meanwhile, it is argued that many practices did not do well in terms of environmental protection, although China has turned to comprehensive land consolidation in terms of policies and ideology, for which food security seems to be the main goal of land policy in China, rather than promoting ecological protection [59,60].

#### 3.2.2. Production Technologies

At first, traditional agriculture focused on improving resource utilization efficiency and intensification to increase domestic food supply, to satisfy the diverse agri-food demand, resulting in high environmental costs and large-scale resource pressures. It is less likely that “high input–high output” farming will be the future of China’s agriculture. After experiencing severe climatic warming and resources limitations, China has begun to explore a sustainable agricultural transformation, based on the principles of “high crop productivity and high resource-use efficiency” (that is, the so-called “double-high” technology system) since 2005 [61], setting national mitigation targets to reach peak carbon dioxide emissions before 2030 and as a means to achieve carbon-neutrality by 2060 [62]. The innovative trajectories of “double-high” agriculture have effectively improved the soil quality, nutrient use efficiency, and adaptation to climate change. As food security is and will be part of the primary agenda, research and policies have shown a gradual emphasis on environmental protection and ecological diversity.

Firstly, much effort has been made in past decades to increase the soil carbon content, including crop straw incorporation, conservation farming, and organic fertilizer application. Soil organic carbon content has increased by 1.5–2 g·kg^−1^ in most cropping areas in China except the northeast, which means that Chinese success in grain production has also contributed greatly to carbon emission mitigation by improving soil fertility during the past two or three decades [62]. Secondly, a series of technological and policy innovation in nutrient management have been developed since 2004 to increase nutrient-use efficiency in China’s grain production [63], including the Zero Increase Action Plan, a project comprising 10,000 mu (≈667 ha) high-yield demonstration areas, farmland protection and quality improvement project, etc. The increase in grain production in China indicates a shift from a quantity-driven, highly resource-dependent, extensive development model to a sustainable intensification model [64]. Thirdly, the Chinese government proposed a new agricultural pattern, namely, Climate-Smart Agriculture (CSA). This was promoted by the FAO to develop agricultural strategies for sustainable food security under the threat of climatic warming, involving policy and mechanism creation, technology integration and demonstration, and institutional capacity-building. China has carried out a large-scale demonstration of the application of climate-smart agriculture in its main grain-producing areas. In the project areas, grain production has increased by more than 5%, with the soil organic carbon content of farmland increasing by 10% [65].

#### 3.2.3. Organization Mode

Existing literature has discussed repeatedly the disconnection and limitations of smallholders, focusing on the causes, necessity, and production vulnerability of a small-scale peasant economy, exploring the path needed for smallholders to integrate into a sustainable agri-food system from political, economic, and ecological perspectives. Several social innovations concerning the smallholder gap between urban markets and rural regions have been developed, to rebuild a more inclusive and just food system [66]. In recent decades in China, new operators of agriculture, including family farms, cooperatives, and “dragon-head” enterprises are emerging. They have the potential to construct large-scale and intensive production bases, disseminate technology to farmers to a large extent through a radiation-driven system, and obtain more market information and policy support [67], although many scholars consider that smallholders are concealed by the capitalist markets that differentiate, destabilize, and transform the meaning of household production [38].

It is clear that e-commerce, as a new mode of alternative food network (AFN), has become a new and effective way to help smallholders to get access to markets. The meteoric rise of “Taobao villages”, representing highly concentrated rural e-commerce centers, has been enabled in part by land consolidation projects [68]. With the implementation of a national rural development project since 2013, a series of government-support measures, like the provision of relevant village-level training courses, have been tackling the problem of connecting smallholders to the market, so as to reduce poverty and rural inequality [69], to improve vulnerable groups’ green credentials and safe production [70], and to make a revolutionary change to the supply chain through eliminating profit-sapping intermediaries.

Since smallholders have a willingness to protect themselves from the lack of food safety from an increasingly fragmented food system, a “bottom-up force of social self-protection” has gradually developed [71]. Some rural producers have adopted a “one family–two system” approach to farming. Agricultural outputs, produced by conventional methods relying on intensive agrochemicals, are usually sold to the market while those cultivated using organic fertilizers (e.g., manure) are left for self-consumption [72]. This represents an emerging folk practice in China’s food system transformation, to ensure food security and food safety. These changes need policy support for organizing farmers to connect with larger consumer groups in the future.

Smallholders play multiple roles in China’s food system. Firstly, smallholders have a resilient social foundation as an essential provider of domestic food output. Secondly, the low income of rural households, caused by inefficient agricultural benefits, restricts their access to adequate, nutritious, and healthy food. Considering the specific characteristics of smallholders in the agri-food system, it is important to explore an efficient mechanism to integrate them, to improve production efficiency and household income. In recent years, this topic has been gradually linked with emerging technology, such as the Internet and e-commerce. Thirdly, there has also been a spontaneous reform force from bottom to top, creating a positive economic and ecological impact. In the future, the digital gap, connection mechanisms, and risk management may be an important field of research, and the role of government policy in the connections among various subjects will also become an explorable theme, to find ways for smallholders to bridge the gap.

#### 3.2.4. Food Reserves

The Chinese government has built food reserve systems consisting of a central reserve and a local reserve with different functions. The central grain reserves are designed to satisfy the basic needs of consumers, to respond to disasters and wars; this is the “ballast stone” of national food security. The national reserves can generally meet demotic food consumption for one year [73]. Local grain reserves are expected to counter emergencies in the regional market, stabilize grain prices, and guarantee supply. Meanwhile, China has an established processed grain reserve system for emergency supply for 10 to 15 days in large and medium-sized cities and areas that are prone to price fluctuations; this covers an emergency reserve, processing, and distribution system [74]. Since the implementation of minimum purchase prices and temporary purchase and storage policies in 2004, a modern grain reserve system integrating central, local, and social resources has been established, which can not only resist the fluctuation of grain prices but also calmly deal with natural disasters and the global food crisis.

The food reserve and circulation systems in China have shown flexibility and effectiveness when responding to major events. Since the COVID-19 pandemic, China has established a coordination mechanism to ensure food supply that involved nine provinces and 500 enterprises for prioritizing the shipment of supplies in times of emergency. The mechanism involved coordination between the central government and local governments, and joint actions by the government and private enterprises [75]. It boosted the supply of grain and cooking oil, released central government reserves of frozen pork, and raised the vegetable supply capacity in some provinces [76]. Local governments launched emergency reserves to further expand the scale of grain reserves by coordinating the transfer of other cities’ grain resources, supporting the resumption of grain enterprises, encouraging enterprises to expand production capacity, and increasing stocks. The government also set up a “green channel” for food transportation, to resolve the difficulties caused by “lockdown” affecting grain transportation. Other measures, e.g., food monitoring systems, public opinion guidance, and temporary subsidies were implemented to guarantee food consumption and ensure food safety in terms of epidemic prevention [77].

The food reserve policy reflects China’s top-down control and management of food security. It is a key means by which to solve the problem of the imbalance between provincial production and demand and the internal food shortage. Many researchers have deconstructed the behavior, layout, and structure of food reserve systems for the reflection and optimization of policymaking, mostly based on qualitative analysis [78,79]. The security of the food supply chain has suffered from multiple challenges, such as natural disasters, health events and policy intervention, highlighting the importance of a reasonable reserve scale and the smooth circulation of the food system. At the same time, cooperation in the whole supply chain will become more complex, requiring multidisciplinary methods to explore risk-response strategies, and the quantitative analysis of food reserve scales and ecological impact needs to be incorporated into the analysis framework of the food system.

#### 3.2.5. Trade Governance

Changes in the diet of residents may lead to a large gap between supply and consumption in feed grain [80], while the global food trade provides an opportunity to alleviate the pressure [81]. In 2013, the Chinese government established a national food security strategy, characterized by self-sufficiency, based on domestic grain production, guaranteed food production capacity, moderate imports, and technological support. Since 2015, China has signed agricultural cooperation agreements with 86 countries along the “Belt and Road”, and has invested in more than 820 agricultural projects. The total investment stock was more than USD 17 billion, and the total agricultural trade within the co-construction countries was around USD 95.79 billion in 2020 [82].

Among the countries along the “Belt and Road”, Russia, Ukraine, Kazakhstan, Romania, Bulgaria, and other emerging grain exporters are not under the comprehensive control of international grain traders. China has strengthened its in-depth cooperation with these countries in all aspects of the industrial chain, including breeding, processing, warehousing, and logistics, etc. The Chinese government has also participated in the construction of its food industry systems and supply chains through investment, trade, and technology transfer [83]. China has included trade in services within the category of “comprehensive agricultural trade”, providing developing countries with full-cycle solutions for industrial chains through foreign aid channels, and realizing dislocation competition with multinational giant companies [84].

Many studies have examined the economic and ecological impacts of the moderate import strategy of China. They suggest moderately reducing the pursuit of a high self-sufficiency rate in terms of food, because this reduces the environmental cost of domestic production and gives consumers more diverse and low-cost food. To protect the safety of the global supply chain while expanding food imports, many researchers have focused on location choices, the layout optimization of global investment, and the “Belt and Road” cooperation promotion strategy. Through practices that include taking part in the initiatives of international organizations, promoting international food regulations, and providing international food aid, China’s role in world food security governance has changed from that of a passive participant to an active builder, which has played a positive role in promoting the transformation of world food security governance in a direction more appropriate for developing countries.

#### 3.2.6. Food Consumption

Food waste and loss have become a matter of public concern because they have led to nutrient losses and the inefficient use of resources, including farmland, energy, water, and fuel for food production [85]. Xue et al. found that 17% of food loss and waste was generated at the consumption stage, of which 77% occurs in out-of-home activities; these figures are much higher than those in industrialized countries. One possible reason is the increase in dining out in China. They also pointed out that halving food waste at the consumption stage would reduce almost the same amount of GHG emissions as halving food loss and waste from the production to retail stage [45]. In responding to food waste and loss, China has taken several measures. For example, the Chinese Anti-Food Waste Law has been issued and was implemented in April 2021. More recently, an action plan was issued in November 2021, proposing that food should be saved throughout the entire food chain and establishing a standard and a monitoring system for reducing food loss and waste. However, compared with the food production section, Chinese policies and practices have paid less attention to the trend of increasing nutrition and healthy demand in terms of food consumption, which is a major source of food waste [86]. There are a number of studies on food waste in China from the macro-level that have assessed its economic and environmental impacts, while few studies have been conducted at the household level. These micro-level studies suggested that educating consumers to foster a green diet and improving dietary knowledge would help to reduce chronic disease and food waste [87,88]. There are several challenges in reducing food waste, apparent from international experiences. These are food waste measurement, quantifying the benefits and costs, examining external connections in the food value chain, and learning how to balance the trade-offs between efficiency, food security, and environmental objectives [89]. Considering that the study of food waste may be a good way to combine production and consumption for systematic research, it is necessary to integrate multidisciplinary methods from fields such as geography, resources and the environment, economy, and society, etc. Lastly, including factors such as institutional design, economic behavior, technical constraints, and social cognition would be helpful in future research to examine the underlying reasons behind food waste.

As China’s food production and consumption systems have rapidly transformed, sustainable food consumption has become a key issue of global concern [90]. The problem of unreasonable dietary structure has gradually been exposed, while the upgrading of food demand from residents has led to the concept of “hidden hunger”, from the perspective of nutrition [73]. Food availability does not guarantee its access [91], which also depends on various factors, such as food allocation, intra-household behavior, and individual food preferences [92]. Thus, anthropometry and household observation aimed at nutritional monitoring can be regarded as an essential supplement of food security measurement. To effectively promote nutritional improvement, China has launched a series of nutrition improvement projects, including nutrition intervention for children and infants, popularizing nutrition knowledge, and supporting the nutrition industry [93]. The heights and weights of children in the pilot areas of the nutrition improvement projects in 2019 have increased significantly, according to the Chinese Center for Disease Control and Prevention [94]. Overall, China still pays more attention to food quantity security, but there is a lack of pursuit of inclusiveness and sustainability on the consumption side compared to developed countries, although in recent years, some scholars have called for more attention to be paid to nutritional health and dietary structure at the household level, to establish a nutrition-oriented food production system and a healthy dietary structure [90]. Therefore, many issues, such as food supply and demand aimed at an overall nutritional goal, the trade-off between food quantity security and nutrition orientation, and long-term nutrition monitoring at both household and individual levels, need to be further studied.

### 3.3. China’s Food Security Achievements and Food System Transformation

**Figure 2:**
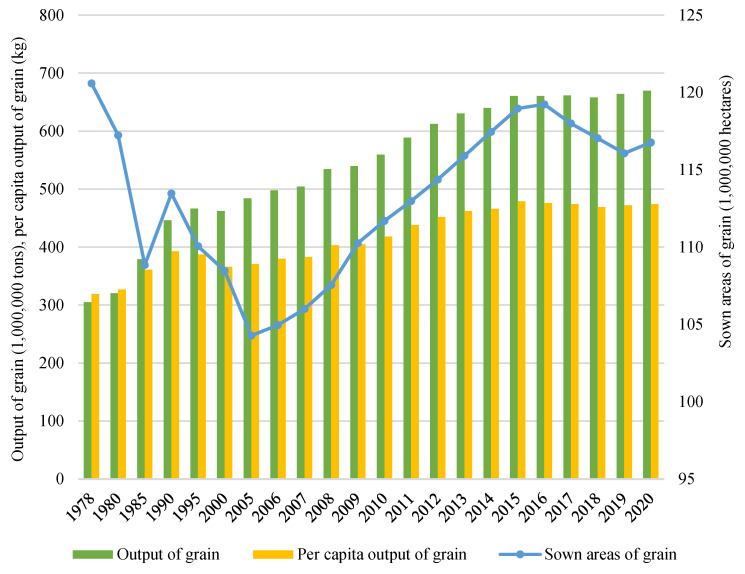
China’s output of grain in 1978–2020. Data source: China Statistical Yearbook.

**Figure 3:**
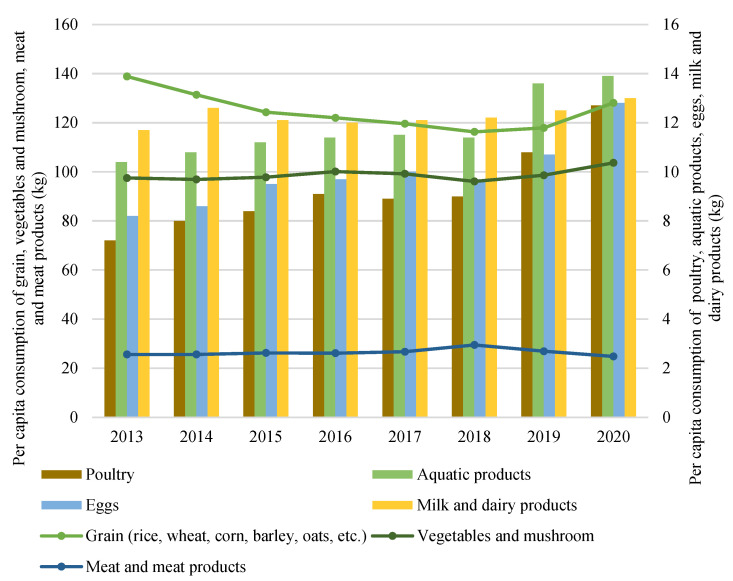
China’s per capita consumption of major foods in 2013–2020. Data source: China Statistical Yearbook.

China’s food security has made great progress in terms of four dimensions: availability, accessibility, affordability, and stability. Firstly, China has achieved self-sufficiency in grain supply in a relatively short period. In 2020, its total grain output was 2.2 times more than that in 1978 (Figure 2). Per capita grain output was around 474 kg, well above the world average of 400 kg [95]. Secondly, the per capita consumption of grains (rice, wheat, corn, barley, oats, etc.) in 2020 was 128.1 kg, approximately 7.8% less than that in 2013, while the per capita consumption of poultry, eggs, aquatic products, and milk and dairy products in 2020 had increased by 76.4, 56.1, 33.7 and 11.1%, respectively (Figure 3). The average daily energy intake of an adult was 2,172 kcal, and the intakes of protein, fat and carbohydrate were 65 g, 80 g, and 301 g, respectively, according to the National Health Commission’s standard. Thirdly, remarkable achievements have been realized in terms of targeted poverty alleviation in China. The per capita disposable income of the rural poor increased from 6,079 yuan in 2013 to 12,588 yuan in 2020, with an annual increase rate of 11.6% [96], making food in the market more affordable. Lastly, extensive (mal)nutritional improvement programs implemented in poor areas have contributed tremendously to the improvement of local residents’ nutrition and health conditions [95].

At present, the transformation of China’s food system is still in the exploratory stages, with certain characteristic principles:

(1) Health and nutrition. The supply-side reform promotes healthier and nutritious food, according to the changing demand of residents, and supplements the shortage of diverse agri-food through international trade and investment;

(2) Inclusivity and fairness. Poverty alleviation policies improve the ability of the poor to obtain food and expand the employment opportunities of vulnerable small farmers through skills training, knowledge studies, and the cultivation of new subjects, so as to ensure the fairness and justice of food security on both the food supply side and the demand side;

(3) Sustainability in all dimensions. China has reduced carbon emissions and optimized the allocation of global resources through ecological innovation, the reduction of food loss and waste, and international resources usage to promote agricultural green development;

(4) Diversity and resilience. China’s food systems have been reoriented around the principles of diversity, multi-functionality, and resilience, to sustain yields and agro-ecosystems in the longer term, and support diverse and nutritious diets;

(5) Social and technological innovation. China’s eco-technological innovation has provided more healthy and nutritious food, and the development of digital agriculture provides opportunities for small farmers to innovate their business models.

An agri-food system transformation around these principles can gradually ensure sustainability in environmental, health, social, cultural, and economic dimensions, and will eventually reinforce the ability to support national food security.

## 4. Conclusions and Recommendations

This paper summarizes China’s food security practices from the perspective of the supply chain, including land consolidation, production technology, organization modes, food reserves, trade governance and food consumption. The experiences and lessons to be drawn from these policies may contribute to the transformation of the global agri-food system in the following ways:

(1) Top-down and bottom-up forces are collectively shaping the transformation of China’s agri-food system as a whole. The standards in the utilization and allocation of agricultural resources, established through top-down power, have set the baseline for domestic food supply. The bottom-up reform force, involving the spontaneous evolution of business models, benefit mechanisms and alternative markets, has increased the possibilities of realizing food safety and environmental sustainability;

(2) China has significantly strengthened its food security and ecological sustainability through expanding food imports. An external food supply eliminates some negative ecological impacts on agricultural production but also increases risks from foreign markets and global economic crises. Therefore, it is crucial for developing countries to improve the efficiency of foreign agricultural investment and participate in global policymaking;

(3) The vulnerability of the global supply chain exposed by the COVID-19 pandemic enlightens us as to the necessity of improving food reserves and rapidly responding to a sudden shock. The food supply in China during the COVID-19 pandemic offers a good demonstration that the resilience of the agri-food system matters.

At present, China’s food system still faces many challenges, including resource and environmental pressure from, e.g., groundwater overexploitation; pesticide and fertilizer overuse; socio-economic challenges, for instance, smallholders having less motivation to grow grain and the lack of innovativeness in the seed industry; trade challenges, such as pressure from international public opinion and tighter import and export regulations. These challenges have directly and/or indirectly led to malnutrition and an unsustainable and inefficient food system. To maximize the potential of agri-food system transformation, more efforts are suggested regarding further studies: (1) to seek integrated solutions through holistic and systematic thinking; (2) to conduct multi-disciplinary cooperation from economics, ecology, politics, geography and other disciplines; (3) to design multi-scale research, e.g., a global-national-local-individual approach is needed to identify the potential challenges and outcomes; (4) to take the mutual support of policy innovation and civil reform into consideration, since the decision-making of actors and power relationships in the system should not be neglected.

## Data Availability

The public datasets analyzed in this study can be found here: http://www.stats.gov.cn/tjsj/ndsj/2021/indexch.htm (accessed on 1 January 2022).
